# Lipoteichoic acid induces surfactant protein-A biosynthesis in human alveolar type II epithelial cells through activating the MEK1/2-ERK1/2-NF-κB pathway

**DOI:** 10.1186/1465-9921-13-88

**Published:** 2012-10-03

**Authors:** Feng-Lin Liu, Chi-Yuan Chuang, Yu-Ting Tai, Hsiu-Lien Tang, Tyng-Guey Chen, Ta-Liang Chen, Ruei-Ming Chen

**Affiliations:** 1 Department of Anesthesiology, Taipei Medical University-Wan Fang Hospital, Taipei, Taiwan; 2 Division of Pulmonology, Department of Internal Medicine, Ren-Ai Branch, Taipei City Hospital, Taipei, Taiwan; 3 Department of Rehabilitation, Po-Jen General Hospital, Taipei, Taiwan; 4 Anesthetics and Toxicology Research Center, Taipei Medical University Hospital, Taipei, Taiwan; 5 Graduate Institute of Medical Sciences; Center of Excellence for Cancer Research, Taipei Medical University, Taipei, Taiwan; 6 Cell Physiology and Molecular Image Research Center, Taipei Medical University-Wan Fang Hospital, Taipei, Taiwan

**Keywords:** Lipoteichoic acid, Alveolar epithelial cells, Surfactant protein-A, MEK/ERK/NF-κB

## Abstract

**Background:**

Lipoteichoic acid (LTA), a gram-positive bacterial outer membrane component, can cause septic shock. Our previous studies showed that the gram-negative endotoxin, lipopolysaccharide (LPS), could induce surfactant protein-A (SP-A) production in human alveolar epithelial (A549) cells.

**Objectives:**

In this study, we further evaluated the effect of LTA on SP-A biosynthesis and its possible signal-transducing mechanisms.

**Methods:**

A549 cells were exposed to LTA. Levels of SP-A, nuclear factor (NF)-κB, extracellular signal-regulated kinase 1/2 (ERK1/2), and mitogen-activated/extracellular signal-regulated kinase kinase (MEK)1 were determined.

**Results:**

Exposure of A549 cells to 10, 30, and 50 μg/ml LTA for 24 h did not affect cell viability. Meanwhile, when exposed to 30 μg/ml LTA for 1, 6, and 24 h, the biosynthesis of SP-A mRNA and protein in A549 cells significantly increased. As to the mechanism, LTA enhanced cytosolic and nuclear NF-κB levels in time-dependent manners. Pretreatment with BAY 11–7082, an inhibitor of NF-κB activation, significantly inhibited LTA-induced SP-A mRNA expression. Sequentially, LTA time-dependently augmented phosphorylation of ERK1/2. In addition, levels of phosphorylated MEK1 were augmented following treatment with LTA.

**Conclusions:**

Therefore, this study showed that LTA can increase SP-A synthesis in human alveolar type II epithelial cells through sequentially activating the MEK1-ERK1/2-NF-κB-dependent pathway.

## Background

Sepsis can lead to multiorgan failure and death and appears to be triggered by bacterial products, such as lipopolysaccharide (LPS) from gram-negative bacteria and lipoteichoic acid (LTA) from gram-positive ones [1-3]. Infection of the respiratory tract caused by gram-positive bacteria and pneumonia combined with acute lung injury (ALI) are usually the leading causes of mortality by sepsis [4]. In the past few decades, the incidences of sepsis and septic shock have been increasing [5]. Although endotoxin-activated events are clearly important in gram-negative infection, gram-positive bacteria also have crucial roles, but less is known about host responses to them [6]. The increasing prevalence of sepsis from gram-positive bacterial pathogens necessitates a reevaluation of the basic assumptions about the molecular pathogenesis of ALI.

Alveolar epithelial type II cells contribute to the maintenance of mucosal integrity by modulating the production of surfactants [7]. Pulmonary surfactants play important roles in protecting the lung during endotoxin-induced injury and infection [8,9]. Surfactant protein (SP)-A is the most abundant pulmonary surfactant protein. Levels of SP-A in bronchiolar lavage fluid are modulated in gram-negative or -positive bacteria-caused lung diseases, including severe pneumonia, acute respiratory distress syndrome, and cardiogenic lung edema [10]. Thus, altering lung SP-A levels can be an effective indicator for pulmonary infection and inflammation. Our previous study showed that LPS selectively induced *spa* gene expression in human alveolar epithelial A549 cells [11].

LTA, an outer membrane component of gram-positive bacteria, was shown to be one of the critical factors participating in the pathogenesis of sepsis [12,13]. LTA can stimulate inflammatory responses in the lung [14,15]. Therefore, understanding the mechanisms that regulate LTA-mediated cell activation is crucial for diagnosis, treatment, or prognosis of lung inflammatory diseases. In response to stimuli, LTA can activate macrophages to produce massive amounts of inflammatory factors that exhibit systemic effects in the general circulation [16]. LTA can induce the secretion of various cytokines such as interleukin (IL)-1β, IL-6, and tumor necrosis factor (TNF)-α [17]. These data suggest that LTA can selectively modify gene transcription of various cell types and sequentially augment and possibly initiate tissue inflammation.

Mitogen-activated protein kinases (MAPKs) are serine/threonine kinases. The first MAPK isoforms to be cloned and characterized were the extracellular signal-regulated kinase 1 and 2 (ERK 1/2) [18,19]. ERK 1/2 are well documented to be activated by a family of dual-specificity kinases known as the mitogen-activated/ERK kinases (MEKs) [16,20]. A previous study demonstrated that LTA can selectively activate the ERK pathway in the cornea [21]. Our previous study showed that LTA induced TNF-α and IL-6 expressions by means of stimulating phosphorylation of ERK1/2 in macrophages [16]. In addition, LTA also triggered translocation of nuclear factor (NF)-κB from the cytoplasm to nuclei and its transactivation activity. Meanwhile, the mechanisms responsible for LTA-induced *spa* gene expression in alveolar epithelial cells are still unknown. In this study, we attempted to evaluate the effects of LTA on SP-1 synthesis in human alveolar type II epithelial cells and its possible mechanisms.

## Materials and methods

### Cell culture and drug treatment

A human lung carcinoma type II epithelial cell line (A549) was cultured following a previous method [3]. A549 cells were grown in Dulbecco's modified Eagle's medium (DMEM)/Ham’s F12 culture medium (Sigma, St. Louis, MO, USA), supplemented with 10% (v/v) heat-inactivated fetal calf serum, 100 U/ml penicillin G, 100 μg/ml streptomycin, and 2 mM l-glutamine. A549 cells were seeded in 75-cm^2^ culture flasks at 37 °C in a humidified atmosphere of 5% CO_2_. Cells were grown to confluence before drug treatment. LTA purchased from Sigma was dissolved in phosphate-buffered saline (PBS) (0.14 M NaCl, 2.6 mM KCl, 8 mM Na_2_HPO_4_, and 1.5 mM KH_2_PO_4_). BAY 11–7082, an inhibitor of NF-κB activation, was also purchased from Sigma.

### Assay of cell viability

Cell viability was determined using a colorimetric 3-(4,5-dimethylthiazol-2-yl)-2,5-diphenyltetrazolium bromide assay as previously described [22]. Briefly, A549 cells (10^4^ cells/well) were seeded in 96-well tissue culture plates overnight. After drug treatment, macrophages were cultured in new medium containing 0.5 mg/mL 3-(4,5-dimethylthiazol-2-yl)-2,5-diphenyltetrazolium bromide for a further 3 h. The blue formazan products in the macrophages were dissolved in dimethyl sulfoxide and spectrophotometrically measured at a wavelength of 550 nm.

### Immunoblotting analyses of SP-A, NF-κB, and phosphorylated and non-phosphorylated ERK1/2 and MEK1

Protein levels were immunodetected according to a previously described method [11]. After drug treatment, cell lysates were prepared in ice-cold radioimmunoprecipitation assay buffer (25 mM Tris–HCl (pH 7.2), 0.1% sodium dodecylsulfate (SDS), 1% Triton X-100, 1% sodium deoxycholate, 0.15 M NaCl, and 1 mM EDTA). Protein concentrations were quantified using a bicinchonic acid protein assay kit (Pierce, Rockford, IL, USA). Proteins (50 μg/well) were subjected to sodium dodecylsulfate polyacrylamide gel electrophoresis (SDS-PAGE) and transferred to nitrocellulose membranes. Immunodetection of SP-A and NF-κB was carried out using rabbit polyclonal antibodies against human SP-A and NF-κB (Santa Cruz Biotechnology, Santa Cruz, CA, USA). Cellular β-actin protein was immunodetected using a mouse monoclonal antibody (mAb) against mouse β-actin (Sigma) as the internal standard. These protein bands were quantified using a digital imaging system (UVtec, Cambridge, UK). Phosphorylated ERK1/2 and MEK1 were immunodetected using a rabbit polyclonal antibody against phosphorylated residues of ERK1/2 and MEK1 (Cell Signaling, Danvers, MA, USA). Nonphosphorylated ERK2 and MEK1 were immunodetected as the internal controls (Cell Signaling). Intensities of the immunoreactive bands were determined using a digital imaging system (Wallac Victor 1420, PerkinElmer, Melbourne, Australia).

### Extraction of nuclear proteins and immunodetection

Amounts of nuclear transcription factors were quantified following a previously described method [20]. After drug treatment, nuclear extracts of macrophages were prepared. Protein concentrations were quantified by a bicinchonic acid protein assay kit (Pierce, Rockford, IL, USA). Nuclear proteins (50 μg/well) were subjected to SDS-PAGE and transferred to nitrocellulose membranes. After blocking, NF-κB was immunodetected using a rabbit polyclonal antibody against mouse NF-κB p65 (Santa Cruz Biotechnology). A proliferating cell nuclear antigen (PCNA) was detected using a mouse mAb against the rat PCNA protein (Santa Cruz Biotechnology) as the internal standard. Intensities of the immunoreactive bands were determined using a digital imaging system (Wallac Victor 1420, PerkinElmer).

### Real-time polymerase chain reaction (PCR) assays

Messenger (m)RNA from A549 cells exposed to LTA were prepared for real-time PCR analyses of SP-A mRNA and β-actin mRNA. Oligonucleotides for the PCR analyses of SP-A mRNA and β-actin mRNA were designed and synthesized by Clontech Laboratories (Palo Alto, CA, USA). The oligonucleotide sequences of the upstream and downstream primers for these mRNA analyses were respectively 5'-TGA AAGGGAGTTCTAGCATCTCACAGA-3' and 5'-ACATATGCCTATGTAGGCCTGACTGAG-3' for SP-A mRNA, and 5'- GTCTACATGTCTCGATCCCACTTA A -3' and 5'-GGTCTTTCTCTCTCATCGCGCTC-5' for β-actin mRNA. A quantitative PCR analysis was carried out using iQSYBR Green Supermix (Bio-Rad, Hercules, CA, USA) and the MyiQ Single-Color Real-Time PCR Detection System (Bio-Rad) as described previously [11].

### Statistical analysis

Statistical differences were considered significant when the *p* value of Duncan’s multiple-range test was <0.05. Statistical analysis between groups over time was carried out by a two-way analysis of variance (ANOVA).

## Results

### Toxicity of LTA to A549 cells

Cell morphology and viability were assayed to evaluate the toxicity of LTA to human alveolar epithelial A549 cells. Exposure of A549 cells to 10, 30, and 50 μg/ml LTA for 24 h did not affect cell viability (data not shown). When exposed to 30 μg/ml LTA for 1, 6, and 24 h, the viability of A549 cells was not influenced. Exposure of A549 cells to 30 μg/ml LTA for 1, 6, and 24 h did not alter cell morphology (data not shown).

### LTA-induced enhancement of SP-A biosynthesis in A549 cells

**Figure 1:**
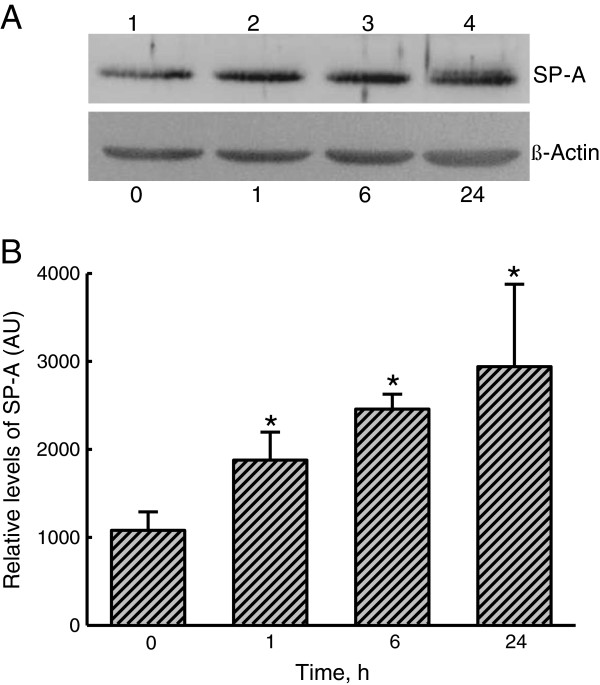
**Effects of lipoteichoic acid** (**LTA**) **on the production of surfactant protein(SP)-A.** A549 cells were exposed to 30 μg/ml LTA for 1, 6, and 24 h (**A**). Cellular proteins were prepared for the immunoblotting analyses. Amounts of SP-A were immunodetected (A, *top pane*). β-Actin was detected as the internal standard (*bottom panel*). These protein bands were quantified and analyzed (**B**). Each value represents the mean ± SEM for *n* = 6. An asterisk (*) indicates that a value significantly differed from the control groups, *p* < 0.05. AU, arbitrary unit.

The effects of LTA on SP-A levels in A549 cells were evaluated by an immunoblotting analysis (Figure 1). In untreated A549 cells, low levels of SP-A were immunodetected (Figure 1A, *top panel*, lane 1). After exposure to 30 μg/ml LTA for 1 h, levels of SP-A were found to be augmented (lane 2). When treated for 6 and 24 h, LTA obviously increased amounts of SP-A in A549 cells. β-Actin was immunodetected (Figure 1A, *bottom panel*). These immunorelated protein bands were quantified and analyzed (Figure 1B). Exposure of A549 cells to 30 μg/ml LTA for 1, 6, and 24 h respectively caused significant 176%, 230%, and 270% increases in SP-A levels.

### LTA-induced SP-A mRNA expression in A549 cells

**Figure 2:**
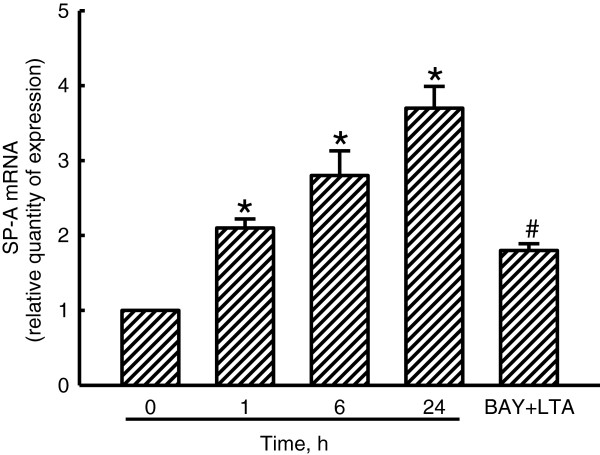
**Effects of lipoteichoic acid (LTA) on induction of surfactant protein(SP)-A mRNA.** A549 cells were exposed to 30 μg/ml LTA for 1, 6, and 24 h. In addition, A549 cells were pretreated with BAY 11–7082 (BAY), an inhibitor of NF-κB activation, for 1 h and then exposed to LTA for another 24 h. mRNA was prepared for real-time PCR analyses of SP-A mRNA and β-actin mRNA. Each value represents the mean ± SEM for *n* = 3. Symbols * and # indicate that the value significantly (*p* < 0.05) differed from the respective control and LTA-treated group, respectively.

Induction of SP-A mRNA expression by LTA was quantified using a real-time PCR analysis (Figure 2). After exposure to LTA for 1 h, the levels of SP-A mRNA in A549 cells were increased by 2.1-fold. Exposure of A549 cells to LTA for 6 and 24 h caused 2.8- and 3.7-fold increases in the levels of SP-A mRNA, respectively (Figure 2). Pretreatment of A549 cells with BAY 11–7082, an inhibitor of NF-κB activation, for 1 h did not change SP-A mRNA expression (data not shown). However, BAY 11–7082 significantly inhibited LTA-induced SP-A mRNA production by 70% (Figure 2).

### Augmentation of NF-κB expression and translocation by LTA

**Figure 3:**
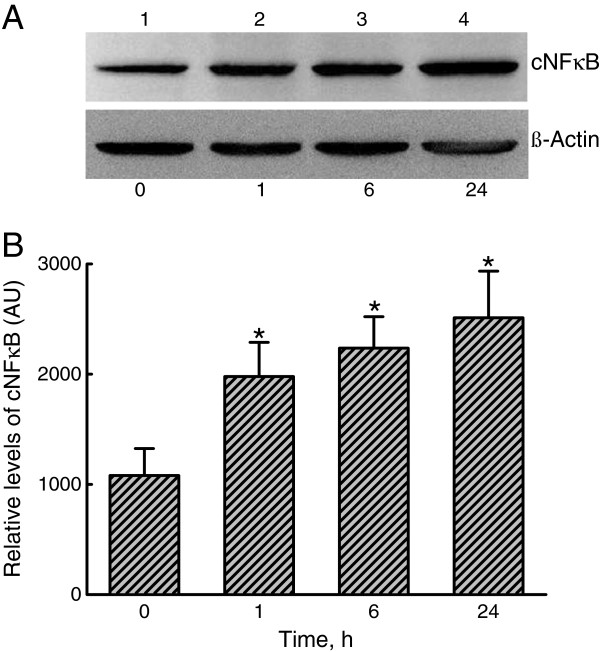
**Effects of lipoteichoic acid (LTA) on the expression of the transcription factor**, **nuclear factor (NF)-κB.** A549 cells were exposed to 30 μg/ml LTA for 1, 6, and 24 h. Levels of cytosolic NF-κB p65 (cNF-κB) were immunodetected (**A**, *top panel*). β-Actin was detected as the internal standard (*bottom panel*). These protein bands were quantified and analyzed (**B**). Each value represents the mean ± SEM for *n* = 6. An asterisk (*) indicates that a value significantly differed from the control groups, *p* < 0.05. AU, arbitrary unit.

**Figure 4:**
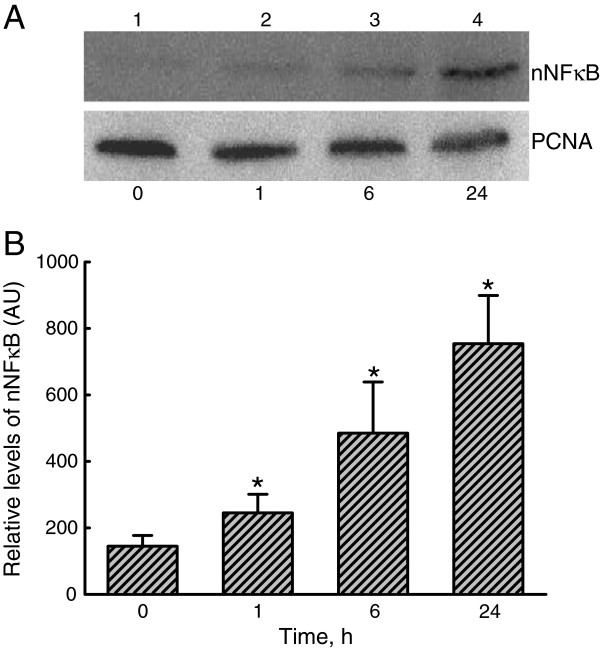
**Effects of lipoteichoic acid (LTA) on translocation of the transcription factor**, **nuclear factor (NF)-κB**, **from the cytoplasm to nuclei.** A549 cells were exposed to 30 μg/ml LTA for 1, 6, and 24 h. Amounts of nuclear NF-κB p65 (nNF-κB) were immunodetected (**A**, *top panel*). Proliferating cell nuclear antigen (PCNA) was detected as the internal standard (*bottom panel*). These protein bands were quantified and analyzed (**B**). Each value represents the mean ± SEM for *n* = 6. An asterisk (*) indicates that a value significantly differed from the control groups, *p* < 0.05. AU, arbitrary unit.

Mechanisms of LTA-induced SP-A augmentation were evaluated by analyses of NF-κB expression and translocation (Figures 3 and 4). Exposure of A549 cells to LTA for 1 h enhanced levels of cytosolic NF-κB (Figure 3A, *top panel*, lane 1). After treatment for 6 and 24 h, the expression of cytosolic NF-κB was obviously augmented (lanes 3 and 4). β-Actin was immunodetected (Figure 3A, *bottom panel*). These immunorelated protein bands were quantified and analyzed (Figure 3B). Exposure of A549 cells to LTA for 1, 6, and 24 h significantly increased NF-κB production by 181%, 200%, and 230%, respectively.

Treatment of A549 cells with LTA for 1 h increased levels of nuclear NF-κB (Figure 4A, *top panel*, lane 2). When exposed for 6 and 24 h, translocation of NF-κB from the cytoplasm to nuclei notably increased (lanes 3 and 4). Amounts of PCNA in A549 cells were immunodetected (Figure 4A, *bottom panel*). These immunorelated protein bands were quantified and analyzed (Figure 4B). Exposure of A549 cells to LTA for 1, 6, and 24 h respectively caused significant 176%, 340%, 530% enhancements in levels of nuclear NF-κB.

### LTA-enhanced phosphorylation of ERK1/2

**Figure 5:**
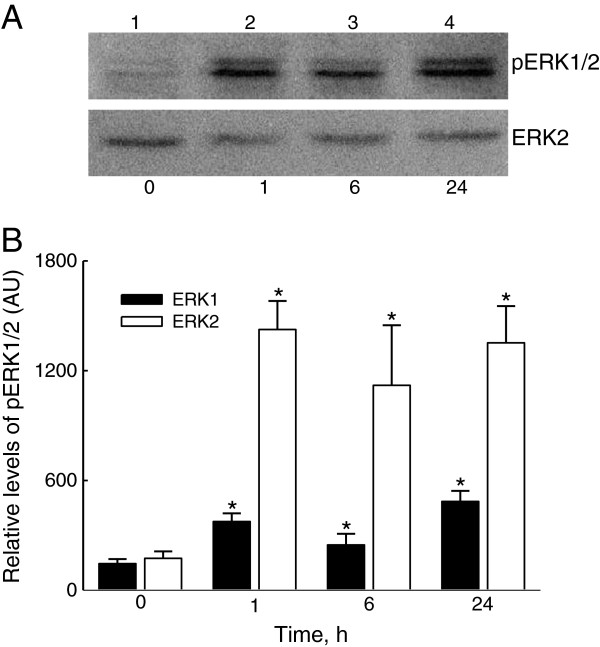
**Effects of lipoteichoic acid (LTA) on the phosphorylation of extracellular signal**-**regulated kinase (ERK)1/2.** A549 cells were exposed to 30 μg/ml LTA for 1, 6, and 24 h. Phosphorylated ERK1/2 (p-ERK1/2) were immunodetected (**A**, *top panel*). ERK2 was detected as the internal standard (*bottom panel*). These immunorelated protein bands were quantified and analyzed (**B**). Each value represents the mean ± SEM for *n* = 6. An asterisk (*) indicates that a value significantly differed from the control group, *p* < 0.05. AU, arbitrary unit.

The reason why LTA improved NF-κB activation was further investigated by assaying ERK1/2 phosphorylation (Figure 5). Treatment of A549 cells with LTA for 1 h increased the amounts of phosphorylated ERK1/2 (Figure 5A, *top panel*, lane 2). Levels of phosphorylated ERK1/2 in A549 cells were obviously raised after exposure to LTA for 6 and 24 h (lanes 3 and 4). Amounts of β-actin in A549 cells were immunodetected (Figure 5A, *bottom panel*). These immunorelated protein bands were quantified and analyzed (Figure 5B). Exposure of A549 cells to LTA for 1, 6, and 24 h significantly increased ERK1 phosphorylation by 259%, 170%, and 334%, respectively. In comparison, levels of phosphorylated ERK2 were respectively augmented by 8.2-, 6.4-, and 7.8-fold following LTA administration for 1, 6, and 24 h (Figure 5B).

### LTA-induced activation of MEK1

**Figure 6:**
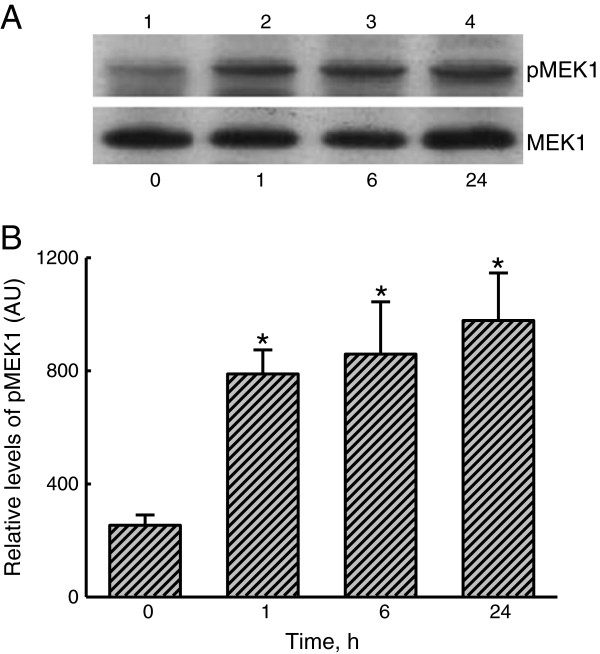
**Effects of lipoteichoic acid (LTA) on the phosphorylation of mitogen**-**activated**/**extracellular signal**-**regulated kinase kinase** (**MEK**)**1**. A549 cells were exposed to 30 μg/ml LTA for 1, 6, and 24 h. Phosphorylated MEK1 (p-MEK1) was immunodetected (**A**, *top panel*). ERK2 was detected as the internal standard (*bottom panel*). These immunorelated protein bands were quantified and analyzed (**B**). Each value represents the mean ± SEM for *n* = 6. An asterisk (*) indicates that a value significantly differed from the control group, *p* < 0.05. AU, arbitrary unit.

Phosphorylation of MEK1 was assayed to determine the mechanism of LTA-induced ERK1/2 activation (Figure 6). Low levels of phosphorylated MEK1 were detected in untreated A549 cells (Figure 6A, *top panel*, lane 1). However, exposure of A549 cells to LTA for 1 h stimulated MEK1 phosphorylation (lane 2). After exposure for 6 and 24 h, the amounts of phosphorylated MEK1 had obviously increased (lanes 3 and 4). β-actin in A549 cells was immunodetected (Figure 6A, *bottom panel*). These immunorelated protein bands were quantified and analyzed (Figure 6B). Treatment of A549 cells with LTA for 1, 6, and 24 h respectively caused significant 82%, 330%, and 370% increases in levels of phosphorylated MEK1.

## Discussion

LTA represents a class of amphiphilic molecules anchored to the outer face of the cytoplasmic membrane in gram-positive bacteria and is commonly released during cell growth, especially under antibiotic therapy [1,2]. It can cause cytokine induction in mononuclear phagocytes [17]. In previous studies, LTA concentrations of 0.2~50 μg/ml were detected and stimulated activity of polymononuclear leucocyte functions and release of TNF-α in peripheral blood mononuclear cells [23,24]. Meanwhile, LTA levels at the infectious site can reach a high level of 26,694 ng/mL [25]. The concentration of LTA used in this study was < 50 μg/ml. Therefore, our results show that LTA at clinically relevant concentrations can activate alveolar type II epithelial cells by stimulating production of surfactants.

During bacterial infection, endotoxins, including LTA and LPS, increase capillary permeability and enhance expressions of cellular adhesion molecules, proinflammatory cytokines, and chemokines [1,15]. These endotoxins can lead to most of the clinical manifestations of bacterial infection and are associated with ALI [4,5]. In addition, LTA can trigger lung inflammation and causes neutrophil influx into the lungs [15,26]. This study showed that in response to LTA stimulation, levels of SP-A mRNA and protein in alveolar A549 cells were time-dependently augmented. SP-A contributes to the pulmonary host defense [10,16,27]. A previous study reported that when *spa* gene expression was knocked-out, susceptibility of the lungs to pathogenic infection was simultaneously raised [28]. Our previous study also showed that LPS-mediated toll-like receptor (TLR) 2 signaling in human alveolar epithelial cells might increase SP-A biosynthesis and subsequently lead to an inflammatory response in the lungs [3]. As a result, SP-A could be an effective biomarker for detecting pulmonary infection by gram-negative or -positive bacteria.

This study showed that LTA increased the expression of NF-κB and its translocation from the cytoplasm to nuclei. NF-κB is a typical transcription factor in response to stimulation by LTA [16]. LTA can bind CD14 and then stimulates TLR activation [16,29]. After LTA associates with TLR2, NF-κB can be activated by protein kinases and is then translocated to nuclei from the cytoplasm [11]. NF-κB regulates certain gene expressions to control cell proliferation, differentiation, and death [30,31]. A previous study showed that LTA induced cyclooxygenase-2 expression in epithelial cells via IκB degradation and successive p65 NF-κB translocation [32]. LTA could induce SP-A mRNA expression in A549 cells. Our bioinformatic search revealed that NF-κB-DNA-binding motifs were found in the promoter regions of the *spa* gene. Suppressing NF-κB activation using BAY 11–7082 simultaneously inhibited LTA-induced SP-A mRNA expression. Thus, LTA transcriptionally induces SP-A expression through inducing NF-κB expression and translocation.

Our present results revealed that the phosphorylation of ERK1/2 was associated with NF-κB activation. Sequentially, ERK1/2-activated IκBα kinase can phosphorylate IκB at two conserved serine residues in the N-terminus, triggering the degradation of this inhibitor and allowing for the rapid translocation of NF-κB into nuclei [16,20]. Accordingly, LTA-induced activation of A549 cells is mainly due to the improvement in ERK1/2 phosphorylation. Roles of ERK1 and ERK2 in LTA-induced SP-A expression were not determined in this study but will be validated using RNA interference in our next study. There is growing evidence that the ERK signaling pathway, which contributes to regulating inflammatory events [33]. Therefore, LTA regulates SP-A expression in alveolar type II epithelial cells in the course of eliciting ERK1/2 phosphorylation and subsequent activation of the transcription factor, NF-κB.

ERK activation is mediated by at least three different pathways: a Raf/MEK-dependent pathway, a PI3K/Raf-independent pathway that strongly activates MEK, and a third undetermined pathway that directly activates ERK proteins [34]. This study showed that LTA time-dependently increased levels of phosphorylated MEK1. Thus, one of the possible reasons explaining why LTA stimulates ERK1/2 activation is the increase in MEK1 phosphorylation. MAPK-regulating signals place this family of protein kinases in an apparently linear signaling cascade downstream of growth factor receptors, adaptor proteins, guanine-nucleotide exchange factors, Ras, Raf, and MEK [19]. The present study demonstrates that LTA can induce SP-A expression via MEK-dependent activation of the protein kinase ERK1/2-signaling pathway.

## Conclusions

In summary, we used an alveolar epithelial cell model to study the immunomodulatory responses of LTA. Our results revealed that LTA can induce inflammatory responses in alveolar epithelial A549 cells by means of enhancing SP-A mRNA and protein syntheses. Moreover, the signal-transducing mechanisms of LTA-caused regulation of SP-A expression arise through the cascade phosphorylations of MEK1 and ERK1/2. In succession, LTA increased NF-κB expression and translocation. LTA-induced SP-A production in alveolar type II epithelial cells may indicate the status of gram-positive bacteria-caused septic shock and acute lung injury. More molecular pathways should be investigated and proven in the future. However, there are certain limitations of this study, including the use of A549 cells, which are derived from human lung carcinoma. The effects of LTA on A549 cells may differ from those on normal alveolar epithelial cells. Thus, we will perform a translational study to evaluate the effects of LTA on alveolar epithelial cells of animals with acute lung injury.

## Abbreviations

ALI: Acute lung injury; ERK1/2: Extracellular signal-regulated kinase 1/2; IL: Interleukin; LPS: Lipopolysaccharide; LTA: Lipoteichoic acid; NF-κB: Nuclear factor-κB; MAPKs: Mitogen-activated protein kinases; MEK1: Mitogen-activated/extracellular signal-regulated kinase kinase 1; PCNA: Proliferating cell nuclear antigen; SDS-PAGE: Sodium dodecylsulfate polyacrylamide gel electrophoresis; SP-A: Surfactant protein-A; TLR2: Toll-like receptor 2; TNF-α: Tumor necrosis factor-α.

## Competing interests

The authors declare that they have no competing interests.

## Authors’ contributions

FLL, CYC, and RMC visualized experimental design. YTT and HLT refined the experimental approach. TGC did the statistical analysis. TLC had significant intellectual input into the development of this work, and added to the Discussion. All authors reviewed data and results, and had significant input into the writing of the final manuscript. All authors read and approved the final manuscript.
